# Successful Treatment and Five Years of Disease-free Survival in a Donor Transmitted Metastatic Melanoma with Ipilimumab Therapy

**DOI:** 10.7759/cureus.4658

**Published:** 2019-05-14

**Authors:** Priyamvada Singh, Deepali Pandey, Brad Rovin, Todd E Pesavento, Thomas Olencki

**Affiliations:** 1 Nephrology and Comprehensive Transplant Center, The Ohio State University Wexner Medical Center, Columbus, USA; 2 Internal Medicine, Saint Vincent Hospital, Worcester, USA; 3 Hematology and Oncology, The Ohio State University Wexner Medical Center, Columbus, USA

**Keywords:** transplant, hla, ipilimumab, ipilimumab, melanoma

## Abstract

Approximately 7% of deceased donors have unknown cancer at the time of organ procurement. More than half of these have no apparent contraindication to organ donation. The commonest transmitted malignancy is renal cell cancer (19%), followed by melanoma (17%). Donor transmission of melanoma has been fatal in most cases as it is commonly metastatic at the time of diagnosis. Till date, there have been only a few cases with remission of melanoma following transplant nephrectomy and withdrawal of immunosuppression. To our knowledge, the evidence presented here is only the second case of donor-derived melanoma that was successfully treated with Ipilimumab. Our patient has the longest disease-free survival (five years) reported in the literature to date.

## Introduction

Allogeneic donor-derived tumors are relatively rare and comprise less than 0.2% of all malignancies in transplant patients. Malignant melanoma is the most lethal of the donor-derived malignancies with an inferior prognosis (less than 5% probability of five-year survival) [1]. The possible explanations for the poor prognosis are the delayed diagnosis of melanoma at metastatic-stage, and lack of equipoise regarding therapeutic management. Till date, there have been only a few cases of remission of melanoma with the withdrawal of immunosuppression, allograft rejection, transplant-nephrectomy, and subsequent systemic chemotherapy. Novel therapy like check-point inhibitors (for instance, Ipilimumab) is changing the landscape of treatment of metastatic melanoma in the general population. However, check-point inhibitors have been successfully utilized only twice in donor-derived melanoma [2,3]. We present here, the longest disease-free survival in a patient with donor-derived melanoma with ipilimumab.

## Case presentation

**Figure 1:**
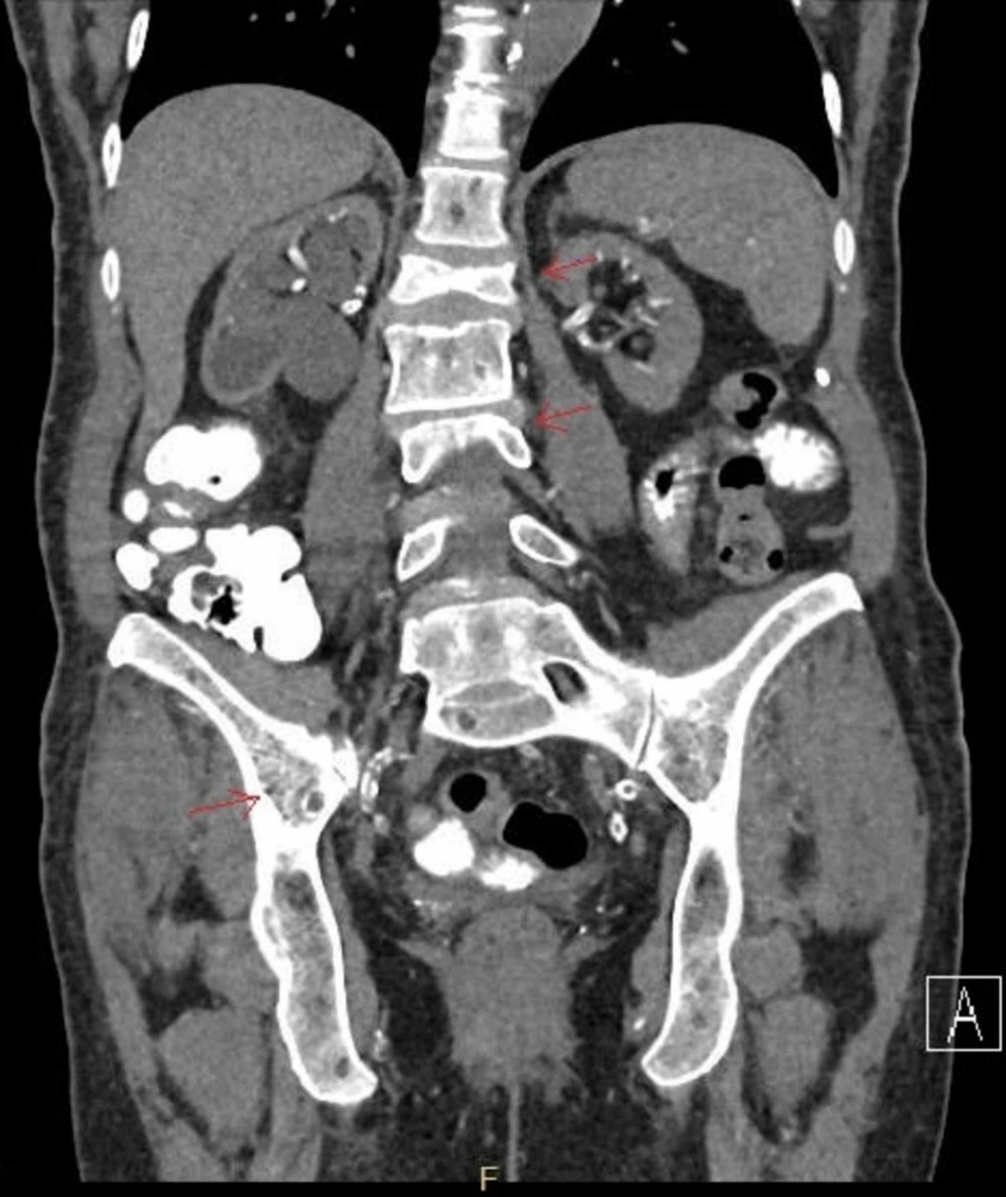
Diffuse osseous metastatic disease with pathologic compression fracture at L1 and L3 is noted, with significant height loss. Diffuse osteopenia is present. Multifocal lytic lesions are present throughout the iliac bones bilaterally.

A 66-year-old female, status-post deceased-donor kidney transplant in 12/2012 for diabetic nephropathy, received a 5/6 (A2, B2, DR1) human leukocyte antigen (HLA) mismatched kidney, induction with basiliximab and glucocorticoid, and maintenance therapy of tacrolimus and mycophenolate. In 03/2013, she developed abdominal pain and acute deterioration of her allograft function. CT of abdomen/pelvis showed pathologic fractures of L1 and L3 and multifocal lytic lesions throughout the iliac bones bilaterally (Figure 1).

**Figure 2:**
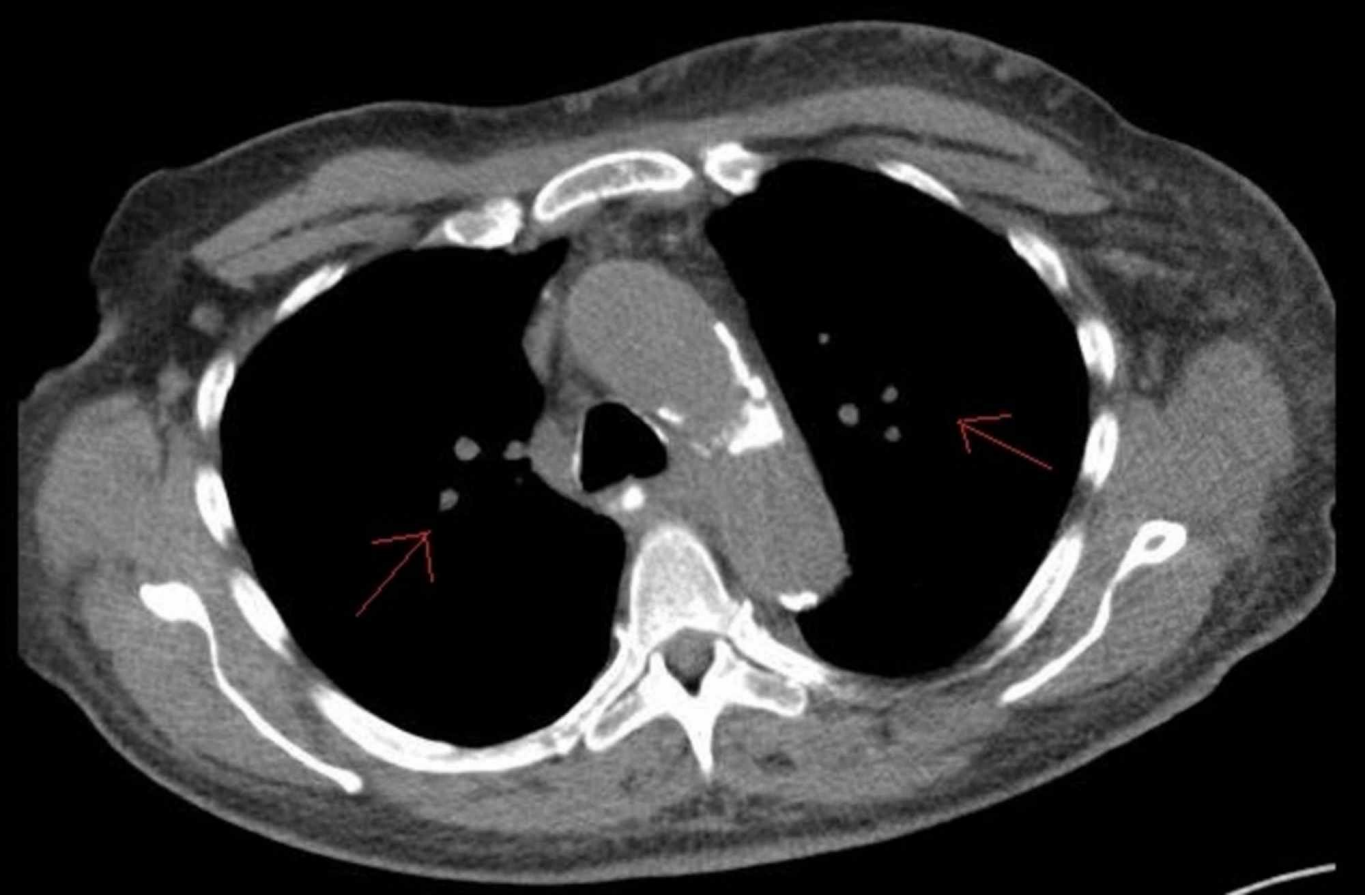
Bilateral scattered small pulmonary nodules.

Biopsy of the posterior iliac spine and sacrum was positive for the involvement of metastatic melanoma. The other organ recipients from the same male donor (partner-kidney and cornea’s recipients) were also diagnosed with donor-derived metastatic melanoma. Hence, it is plausible to say that our patient had melanoma originated from the same source. The HLA typing of the tumor cells demonstrated XY, male-karyotype, further strengthening the diagnosis of donor-derived melanoma in our female patient. Her immunosuppressive medications were discontinued, followed by graft rejection, explantation of the allograft (which showed melanoma), and initiation of dialysis. Staging studies showed metastatic disease involving bone, spleen, and lungs (TxNxM1c stage IV) (Figure 2). Her melanoma showed BRAF-V600E favorable mutation. She was started on vemurafenib (960 mg bid, from 4/2013 to 08/2013). It was later discontinued due to extensive cutaneous lesions. She was switched to Ipilimumab (3 mg/kg, every three weeks from 8/13/13 to 10/15/13, four-cycles in total) without any noteworthy side effects, significant improvement in the quality of life (90% performance status), no further progression of cancer on serial imaging, and complete remission for five years now.

## Discussion

Despite careful donor selection, cancer transmission to the recipient is inevitable [1]. Malignant melanoma is considered to be an immunogenic tumor, which changes its malignant potential with the exposure to immunosuppression [1,2]. Melanoma could remain dormant chronically in the immunocompetent population. Whereas, the immunocompromised environment presents an excellent environment for the development and progression of these quiescent donor’s melanoma. A careful history of the donor including occupational, sun exposure, and family history of cancers should ideally be obtained before organ retrieval. Furthermore, if possible, all donors should undergo an autopsy to exclude the presence of neoplastic disease. Realistically, these are at times not reasonable given the time constraints and organ scarcity. Following the identification of donor-derived metastatic melanoma in a recipient, the outcome of the remaining recipients of multiorgan donations should be actively pursued. Pathological analysis along with tissue-typing laboratory and fluorescent in situ hybridization (FISH) analysis could aid in the diagnosis of donor-derived melanoma.

There is a lack of consensus regarding the management for donor-derived melanoma. Early diagnosis, cessation of immunosuppression to allow rejection of the allograft and transplanted cancer cells, transplant nephrectomy are the usual standards in patients with kidney transplant [1,2]. Unfortunately, this is not a feasible option for patients with transplanted organs other than kidneys (heart, lung, and pulmonary) due to a lack of viable alternative therapies to the transplanted organs. Chemotherapy/interferon that has been used as adjuvant therapy in past few case scenarios has failed to produce promising results. The reported cases of donor-derived melanoma along with their outcomes and treatment are described below (Table 1). For patients with established allogeneic tumors, new approaches to immunotherapy, including cloned alloreactive Cytotoxic T Lymphocytes (CTLs), are emerging as an option. Checkpoint inhibitors block the adaptive inhibitory mechanisms of the immune system and allow immune cells to recognize and eradicate tumor cells. They have provided sustainable disease remission in once incurable cancers immunotherapy, for instance, ipilimumab has once been previously reported to have produced a disease-free survival of 16 months in a donor-derived melanoma [4]. Our patient is the third successful case of remission in donor-derived melanoma with checkpoint-inhibitors, and the very first with a reported five years of disease-free survival. She tolerated ipilimumab well without much adjustments to the dialysis treatment. The degree of antigen-mismatch between the donor and the recipient is suggested as a favorable factor for treatment response [5]. We speculate the five antigen-mismatch might have favored our patient’ antitumor T-cell response. It has been earlier suggested that the success of this treatment approach depends upon the degree of antigen mismatch between a tumor and the recipient; the better results were in those with the higher degree of mismatch which may offer a more reliable and robust way to develop an effective antitumor T-cell response [3, 4]. Future studies regarding mechanistic approach and therapeutics are warranted.

**Table 1:** Successful use of check-point inhibitors for donor-derived metastatic melanoma.

Case	Time of diagnosis post-transplant	Diagnostic clues for donor-derived melanoma	Melanoma mutation targeted therapy	Other treatments	Outcome at time of publication
Chen et al. (2012) [2]	One year	Cerebral metastatic melanoma in hepatic allograft from the same donor, positive imaging studies, and mutations - S100, HMB45, and MELAN-A.	BRAF negative	Bilateral transplant nephrectomy, withdrawal of immunosuppression, rejection of organ, explantation, brain radiation for cerebral metastasis. Ipilimumab (3 mg/kg, four cycles).	Sixteen months with no evidence of disease.
Boyle et al. (2017) [3]	One month	Pan CT-scan done for constitutional symptoms came positive for metastatic lesions. Positive S-100, SOX-10, melanin-A and BRAF-V600E mutation.	Dabrafenib 150 mg, Trametinib 2 mg once daily (stopped due to gastrointestinal intolerance).	Bilateral transplant nephrectomy, withdrawal of immunosuppression, rejection of organ, explantation. Nivolumab (3 mg/kg, every two weeks)	At 14 months, CT imaging demonstrated continued tumor regression.
Singh (2019), the current case	Three months	Renal dysfunction followed with positive CT scan for metastatic lesions. Positive BRAF-V600E mutation, recipient of sister-kidney, and cornea with metastatic-melanoma.	Vemurafenib (960 mg bid) from 4/2013 to 08/2013, discontinued due to extensive cutaneous lesions.	Bilateral transplant nephrectomy, withdrawal of immunosuppression, rejection of organ, explantation. Ipilimumab (3 mg/kg, four cycles)	Five years with no evidence of disease.

## Conclusions

Through our case report, we hope to draw the attention of the medical community regarding immunotherapy as a possible adjunct in the therapy of donor-derived melanoma and reinforce the importance of a detailed history, examination, and autopsy of a deceased donor before organ retrieval. The role of HLA mismatch in prognosis is unclear and a topic for future research.
